# Einarbeitung junger Fachkräfte auf der Intensivstation: Eine bundesweite Onlineumfrage der Jungen DIVI – einer Initiative der Deutschen Interdisziplinären Vereinigung für Intensiv- und Notfallmedizin (DIVI)

**DOI:** 10.1007/s00063-023-01067-y

**Published:** 2023-09-27

**Authors:** David Josuttis, Frida Regner, Teresa Deffner, Diana Freund, Felix Freund, Celina Cornelius, Angelina Beer, Aileen Spieckermann, Matthias Manfred Deininger

**Affiliations:** 1 Klinik für Anästhesiologie, Intensiv- und Schmerzmedizin, BG-Klinikum Unfallkrankenhaus Berlin, Warener Str. 7, 12683 Berlin, Deutschland; 2 Klinik und Poliklinik für Kinder- und Jugendmedizin, Universitätsklinikum Carl Gustav Carus Dresden, Dresden, Deutschland 01304,; 3 Klinik für Anästhesiologie und Intensivmedizin, Universitätsklinikum Jena, Jena, Deutschland Am Klinikum 1, 07747; 4 Klinik für Anästhesie, Intensiv‑, Notfall- und Schmerzmedizin, Universitätsmedizin Greifswald, Greifswald, Deutschland Ferdinand-Sauerbruch-Straße, 17475; 5 Offshore Rescue and Medical Service, Die Johanniter-Unfall-Hilfe e. V., Am Deich 8, 27804 Berne-Bardenfleth, Deutschland; 6 Klinik für Anästhesiologie, Universitätsklinikum Heidelberg, Heidelberg, Deutschland Im Neuenheimer Feld 672, 69120; 7 Chirurgische Universitätsklinik und Poliklinik, BG Universitätsklinikum Bergmannsheil, Bergmannsheil, Deutschland Bürkle de la Camp-Platz 1, 44789; 8 Klinik für Anästhesiologie und Klinik für Operative Intensivmedizin und Intermediate Care, Uniklinik RWTH Aachen, Aachen, Deutschland Pauwelsstraße 30, 52074

**Keywords:** Ankommen, Berufsstart, Zufriedenheit, Nachwuchs, Kompetenz, Induction, Training, Job satisfaction, Junior staff, Skills

## Abstract

**Hintergrund:**

Das Ankommen auf der Intensivstation ist für Fachkräfte entscheidend, um hochqualitative Patient:innenversorgung gewährleisten zu können. Dieser Prozess hängt von der Einarbeitungsqualität ab.

**Ziel der Arbeit:**

Ziel der Arbeit ist die Erfassung von Art und Umfang der Einarbeitung und der Zufriedenheit von jungen Fachkräften auf der Intensivstation.

**Material und Methoden:**

In einer anonymen, berufsgruppenübergreifenden Onlineumfrage wurde die Einarbeitungsstruktur sowie Zufriedenheit untersucht.

**Ergebnisse:**

Von 554 Teilnehmenden kamen etwa zwei Drittel aus dem ärztlichen Bereich. Während bei der Pflege 59 % ein schriftliches Einarbeitungskonzept aufwiesen, lag ein solches nur bei 27 % der Ärzt:innen vor. Pflegefachpersonen gaben im Median 30 Tage als Einarbeitungszeit, Ärzt:innen 7 Tage an. Ein Drittel der Pflegefachpersonen stimmte der Aussage zu, ausreichend durch die Einarbeitung auf die intensivmedizinische Tätigkeit vorbereitet worden zu sein. Im ärztlichen Bereich fühlten sich nur 17 % der Teilnehmenden ausreichend durch die Einarbeitung vorbereitet. Mehr als 42 % der Befragten konnten sich vorstellen, noch länger als 3 Jahre in der Intensivmedizin tätig zu sein.

**Diskussion:**

Evidenzbasierte Methoden zur Strukturierung der Einarbeitung von neuen Fachkräften sind in deutschen Intensivstationen nur gering verbreitet. Die Einarbeitungszeit liegt weit unter publizierten Empfehlungen. Auch wenn bezüglich der materiellen und personellen Ausstattung Defizite wahrgenommen werden, geben die meisten Befragten Zufriedenheit mit Lernzuwachs und interprofessioneller Zusammenarbeit an.

**Zusatzmaterial online:**

Zusätzliche Informationen sind in der Online-Version dieses Artikels (10.1007/s00063-023-01067-y) enthalten.

## Abstract (en)

**Background:**

Onboarding of junior staff in the intensive care unit is vital to ensure high-quality critical care treatment. This process depends on beginner’s training.

**Aim:**

We aimed to determine structure and duration of intensive care onboarding and the job satisfaction of junior professionals in German intensive care units.

**Materials and methods:**

We conducted an anonymous, interprofessional online survey regarding quality of onboarding and job satisfaction among young professionals.

**Results:**

A total of 554 young professionals participated, about two thirds were physicians. A written concept was used in 59% of the nurse’s and 27% of physicians’ training. Median duration of training before taking full charge of patient treatment was 30 days among nurses and 7 days among physicians. About one third of nurses and 17% of physicians stated that they were sufficiently prepared after the training period, whereby 49% of physicians often felt overwhelmed. More than 42% can imagine working in critical care longer than the next 3 years.

**Conclusion:**

Fundamental methods for training of critical care professionals starting their intensive care career are underused in Germany and the duration of training blatantly differs from national guideline recommendations. Although there seem to be deficits concerning material and staff resources, participants are satisfied with learning progress and teamwork.

## Hintergrund

Rund ein Drittel der Intensivbetten war im Oktober 2021 nicht betreibbar, wie eine Umfrage der Deutschen Interdisziplinären Vereinigung für Intensiv- und Notfallmedizin (DIVI) ermittelte. Als primäre Ursache wird in 75 % aller Sperrungen der Fachkräftemangel angegeben [4]. Die Gewinnung und motivierende Einarbeitung von jungen Fachkräften erscheint vor dem Hintergrund des steigenden Personalbedarfs als eine essenzielle Zukunftsaufgabe. Die Komplexität und Schwere der zu behandelnden Patient:innen und Krankheitsbilder kombiniert mit logistischen, technischen und organisatorischen Herausforderungen machen die Intensivmedizin zu einem anspruchsvollen Hochrisikobereich. Es erscheint daher unbedingt erforderlich, dass neue Kolleg:innen die besonderen Prozesse und Abläufe vor einem eigenverantwortlichen Einsatz auf der Intensivstation erlernen. In den DIVI-Strukturempfehlungen zur „Ausstattung und Struktur von Intensivstationen“ aus dem Jahr 2022 (folgend: DIVI-Strukturempfehlungen) wird für die Einarbeitung ein zeitlicher Umfang von 3 Monaten gefordert [8].

Während die berufliche Zufriedenheit in einzelnen Facharztausbildungen auch im intensivmedizinischen Kontext bereits untersucht wurde, ist die fachgebietsübergreifende Zufriedenheit mit der intensivmedizinischen Tätigkeit bisher kein Gegenstand von Untersuchungen gewesen [1, 2]. Deshalb war es Ziel dieser Erhebung, die Situation des Ankommens in der Intensivmedizin berufsgruppenübergreifend quantitativ zu erfassen, um die aktuelle Einarbeitungspraxis in Deutschland bewerten zu können. Weiterhin sollte die aktuelle allgemeine berufliche Zufriedenheit von intensivmedizinisch Tätigen eruiert werden.

## Methode

Zur Beantwortung der Fragestellungen wurde eine anonyme Onlineumfrage durchgeführt. Die Autorengruppe, bestehend aus jungen Fachkräften verschiedener Professionen, leitete anhand der Umfrageziele mit Unterstützung durch eine methodenkompetente Psychologin konkrete Fragen ab. Die Umfrage betonte einen interprofessionellen und interdisziplinären Ansatz und fokussierte auf Ärzt:innen und Pflegefachpersonen, die in der Intensivmedizin tätig waren oder sind.

Zunächst erfolgte eine Literaturrecherche mit dem Fokus auf evaluierte Methoden und Verfahren in der intensivmedizinischen Einarbeitung. Hier zeigte sich, dass insbesondere die folgenden Aspekte bereits als relevante Einflussfaktoren auf das Ankommen in der Intensivmedizin untersucht wurden: 1.) Ein strukturiertes Einarbeitungskonzept erwies sich im ärztlichen Dienst als zufriedenheitsfördernd und qualitätsverbessernd [7]. 2.) Bei Feld et al. zeigten sich ähnliche vorteilhafte Effekte durch die Bestellung eines einarbeitungsverantwortlichen Clinical Mentors für neue Kolleg:innen [3]. 3.) Simulationstrainings bieten überdies die Möglichkeit, sich neuen Situationen in geschütztem Rahmen anzunähern, und sind gerade in kritischen Arbeitsbereichen eine gut validierte Methode [6]. 4.) Ein sog. Intensivvorbereitungskurs fasst theoretische und praktische Lerninhalte für eine Gruppe von neuen Mitarbeitenden zusammen und soll Unsicherheiten abbauen.

Diese Einarbeitungsaspekte können jeweils als empfehlenswert eingeschätzt werden und wurden in einem beschreibenden Eingangstext erläutert sowie die Verfügbarkeit bzw. der Wunsch danach einzeln abgefragt (vollständiger Fragebogen als Material S1 im Supplement).

Inwiefern die eigene Einarbeitung und die persönliche klinische Tätigkeit als zufriedenstellend beschrieben wurde, wurde als Maß der Zustimmung auf einer 5‑stufigen Likert-Skala erfasst. Aufgrund der Fragebogenlogik mit der Möglichkeit von überspringenden Verweisen in Abhängigkeit von Vorfragen unterschied sich die Gesamtzahl der zu beantwortenden Fragen pro Teilnehmer:in. Insgesamt wurden den Teilnehmenden bis zu 33 Fragen gestellt, wobei sowohl Single- als auch Multiple-Choice– sowie Freitextfragen zum Einsatz kamen. Die Beantwortung nahm etwa 10–15 min in Anspruch. Der Fragebogen wurde wiederholt im Autorenteam reevaluiert. Im Anschluss erfolgte ein Pretest auf Konklusivität und Verständlichkeit bei 6 bisher Unbeteiligten. Nach erfolgreichem Pretest wurde der Fragebogen in die Onlineplattform SurveyMonkey (SurveyMonkey Inc., San Mateo, Kalifornien, USA) übertragen. Aufgrund der anonymen Teilnahme sowie der plattformseitigen Einstellung, keine zusätzlichen persönlichen bzw. identifizierbaren (wie etwa IP-Adresse) Daten auszugeben, war vor der Durchführung der Studie keine ethische Beratung erforderlich. Zudem wurde allen Teilnehmenden ein Informationstext über Ziel der Umfrage, durchführendes Gremium inklusive Kontaktmöglichkeit, Art und Umfang der Datenverarbeitung, Freiwilligkeit der Teilnahme und Beantwortung einzelner Fragen angezeigt und erst nach entsprechend erteiltem Einverständnis durch jede:n Teilnehmende:n die Umfrage begonnen. Zunächst erfolgte eine Bewerbung der Teilnahme unter den Mitwirkenden der Jungen DIVI (Initiative innerhalb der DIVI von aktuell ca. 70 jungen Fachkräften und Studierenden mit Interesse an Intensiv- und Notfallmedizin) mit der Bitte, die Umfrage auch im jeweiligen beruflichen Umfeld publik zu machen. Außerdem wurde mithilfe des DIVI-Newsletters (Reichweite circa 14.000 Abonnent:innen) sowie den Social-Media-Auftritten der DIVI zur Teilnahme an der Umfrage eingeladen. Die Umfrage wurde vom 06.10.2022 bis zum 06.11.2022 durchgeführt. Die Ergebnisse wurden in SPSS 28.0 (IBM Corp., Armonk, NY, USA) analysiert. Zeiträume werden als Median mit Interquartilsabstand (IQR) angegeben. Die Auswertung und Zuordnung der Freitextantworten erfolgte jeweils unabhängig durch 2 Autor:innen.

## Ergebnisse

### Beschreibung des Studienkollektivs

Insgesamt konnten 554 Datensätze ausgewertet werden. Hierunter waren 370 Ärzt:innen (67 %) und 184 Pflegefachpersonen (33 %). Eine vollständige Übersicht der demografischen Daten ist in Supplement (Tabelle S2) zu finden. Unter den ärztlichen Teilnehmenden waren 61 % in Weiterbildung. Von den teilnehmenden Ärzt:innen gaben 19 % an, über die Zusatzweiterbildung Intensivmedizin zu verfügen. Bei den Krankenhausstufen der teilnehmenden Ärzt:innen handelte es sich überwiegend um Universitätskliniken (50 %). Die größte Zahl der Teilnehmenden (63 %) war in einer Intensivstation mit 10–20 Beatmungsbetten aktiv. Unter den Pflegefachpersonen verfügten 53 % über eine Fachweiterbildung und 17 % über eine akademische Qualifizierung. Bei den Kliniken der teilnehmenden Pflegefachpersonen handelt es sich überwiegend um Maximalversorger (29 %) oder Unikliniken (36 %). Sie waren mehrheitlich auf Intensivstationen mit 10–20 Betten tätig (67 %). Während von den Pflegefachpersonen 64 % ihre intensivmedizinische Tätigkeit vor 2017 begonnen haben, starteten 54 % der teilnehmenden Ärzt:innen erst ab 2020 auf der Intensivstation.

### Intensivmedizinische Einarbeitung

**Figure 1:**
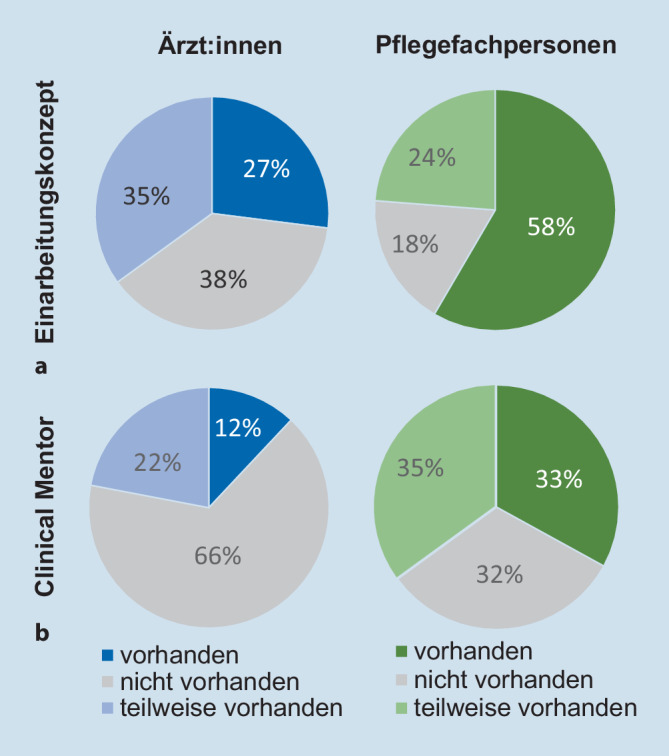

Von den befragten Ärzt:innen gaben 27 % an, über ein Einarbeitungskonzept zu verfügen, während die Quote im pflegerischen Bereich bei fast 60 % lag. Etwa 35 % der Ärzt:innen und 24 % der Pflegefachpersonen konnten das Vorhandensein eines Einarbeitungskonzepts nur teilweise bejahen (siehe Abb. 1a). Als Gründe hierfür wurden unter den Antwortoptionen überwiegend die fehlenden personellen Ressourcen bzw. die fehlende praktische Umsetzung des verschriftlichten Konzepts ausgewählt. Etwa 12 % der teilnehmenden Ärzt:innen war ein (Clinical) Mentor zugeordnet. Bei Pflegefachpersonen betrug die Quote ein Drittel (s. Abb. 1b). Als Gründe für eine Teilanwendung des Clinical-Mentor-Konzeptes (von ärztlich 20 % bzw. pflegerisch 35 % der Teilnehmenden angegeben) wurde die zu geringe Zeit mit dem/der Mentor:in oder die fehlende direkte, persönliche Zuordnung einer Ansprechpartnerin/eines Ansprechpartners angeführt.

**Figure 2:**
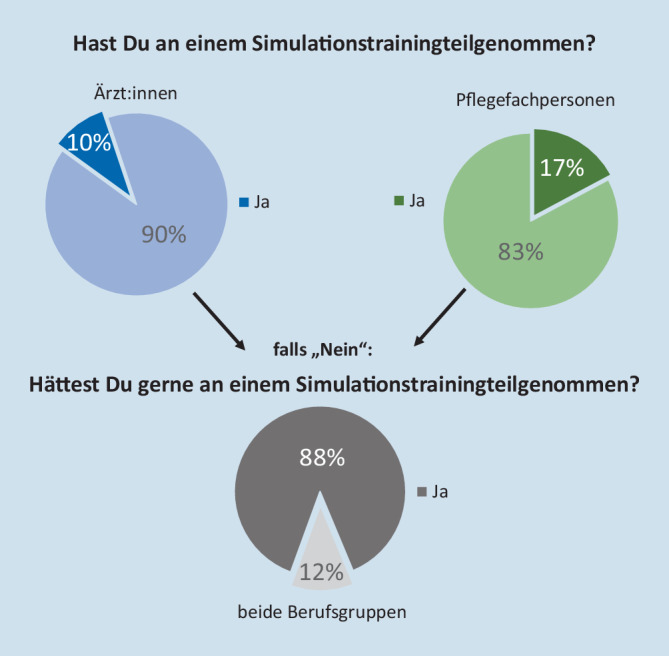

Während 17 % der Pflegefachpersonen ein Simulationstraining erhielten, wurde dies nur von knapp 10 % der Ärzt:innen bejaht. Von den pflegerischen respektive ärztlichen Befragten, die an einem Simulationstraining teilgenommen hatten, empfanden dies 77 % bzw. 71 % rückblickend als hilfreich oder sehr hilfreich. Rund 88 % der Personen, die kein Simulationstraining besuchten, hätten sich ein solches gewünscht (s. Abb. 2). Pflegerisch war 20 % der Teilnehmenden das Konzept eines Intensivvorbereitungskurses bekannt, 7 % davon nahmen an einem solchen teil. Von den Ärzt:innen kannten ca. 46 % das Kursformat und 21 % der Teilnehmenden beteiligten sich auch an einem solchen. Als Grund für die Nichtteilnahme wurde überwiegend der ungünstige Zeitpunkt eines angebotenen Intensivvorbereitungskurses angegeben. Außerdem wurden mangelnde Freistellung oder die Teilnahmegebühren als Teilnahmehindernisse benannt.

**Figure 3:**
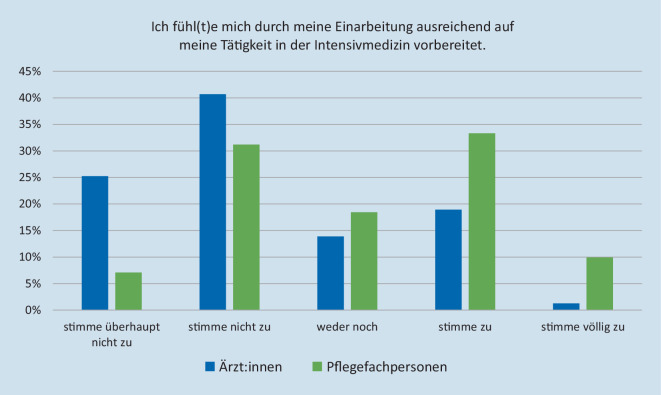

Pflegefachpersonen gaben eine mediane Einarbeitungszeit von 30 Tagen (IQR 20–60) an, der erste eigenverantwortliche Nachtdienst wurde 40 (IQR 25–61) Tage nach Berufsstart angetreten. Ärzt:innen wurden 7 (IQR 3–14) Tage eingearbeitet und waren nach 22 (IQR 14–35) Tagen eigenverantwortlich im Nachtdienst tätig. Ein Drittel der Pflegefachpersonen stimmte der Aussage (völlig) zu, ausreichend durch die Einarbeitung auf die Tätigkeit vorbereitet worden zu sein (siehe Abb. 3). Es gaben 27 % an, sich oft überfordert zu fühlen. Im ärztlichen Bereich stimmten 66 % der Aussage, sich ausreichend durch die Einarbeitung auf die Tätigkeit vorbereitet zu fühlen, (überhaupt) nicht zu. Es fühlten sich 49 % der teilnehmenden Ärzt:innen oft überfordert.

Die Wünsche der Teilnehmenden zu einer zufriedenstellenden Einarbeitung wurden im Rahmen einer Freitextfrage erfasst. Diese wurde von 442 der Teilnehmenden ausgefüllt. Am häufigsten (13 %) wurde angemerkt, dass in der Einarbeitung ein:e feste:r Ansprechpartner:in oder Mentor:in wichtig sei. 6 % der Teilnehmenden wünschten sich gezieltere Vermittlung von theoretischen Grundlagen, teilweise wird hier auch eine Notwendigkeit von Lernzieldefinitionen oder Lernerfolgskontrollen angesprochen, 5 % der Teilnehmenden äußerten den Wunsch nach verbessertem didaktischem Aufbau bzw. bedarfsgerecht angepasster Strukturierung der Einarbeitung. Des Weiteren wurde mehrfach eine verstärkte Präsenz von Ober- oder Fachärzt:innen, frühzeitigere Kommunikation des geplanten Beginns der Intensivrotation, klarere Darstellung von Lernzielen mit Lernerfolgskontrollen sowie eine strukturierte Geräteeinweisung gewünscht. Viele der weiteren Antworten waren von grundsätzlichen Anmerkungen zu Bürokratie, Personalmangel und pandemiebedingten Einschränkungen geprägt.

### Zufriedenheitsanalyse zur intensivmedizinischen Tätigkeit

**Figure 4:**
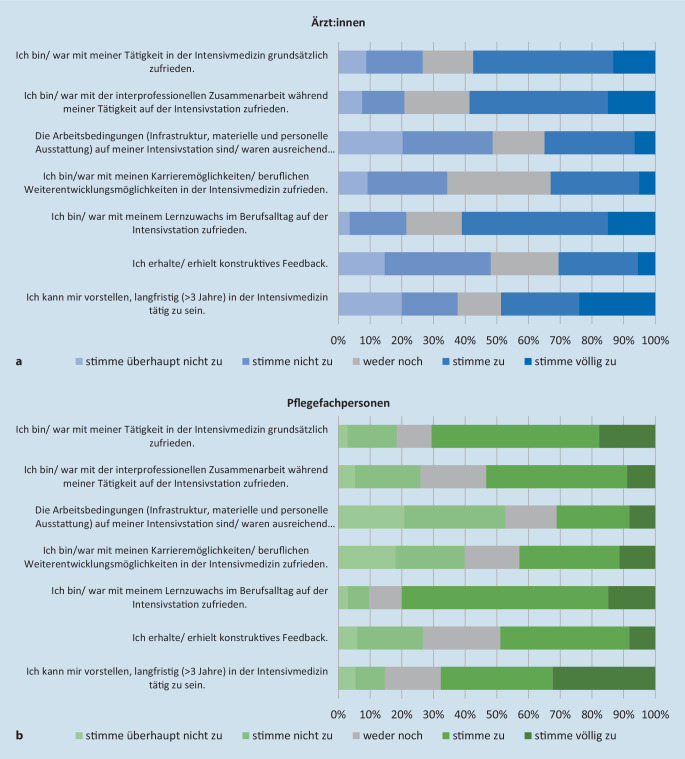

Die Mehrheit der Befragten war mit ihrer Tätigkeit in der Intensivmedizin grundsätzlich zufrieden und mehr als 42 % der Befragten konnten sich vorstellen, noch länger als 3 Jahre in der Intensivmedizin tätig zu sein (s. Abb. 4).

Bezüglich der Arbeitsbedingungen in der Intensivmedizin bejahten 34 % der Teilnehmenden beider Berufsgruppen die Aussage, dass Infrastruktur sowie materielle und personelle Ausstattung ausreichend und zweckmäßig seien. Überwiegend positiv wurde die Kooperation der Berufsgruppen auf der Intensivstation eingeschätzt. Der Aussage, mit der interprofessionellen Zusammenarbeit zufrieden zu sein, stimmten 53 % der Pflegefachpersonen (vollständig) zu. Ähnlich wurde dies im ärztlichen Bereich wahrgenommen. Hier lag die Quote der Teilnehmenden mit (völliger) Zustimmung bei 59 %. Die Frage, ob der eigene Lernzuwachs als zufriedenstellend betrachtet wird, wurde mit großer Mehrheit von etwa zwei Dritteln der Befragten in beiden Berufsgruppen zustimmend beantwortet. Allerdings stimmten 22 % der teilnehmenden Ärzt:innen nicht oder überhaupt nicht zu. Unter den Pflegefachpersonen lag diese Quote bei 10 %. Im ärztlichen Bereich wurde die Aussage, dass konstruktives Feedback gegeben werde, weniger oft bejaht (31 %) als im pflegerischen Bereich (49 %). Die Zufriedenheit mit den Karrieremöglichkeiten wurde in beiden Berufsgruppen auf der Likert-Skala etwa ausgewogen (entsprechend der Antwortoption „weder noch“) bewertet.

## Diskussion

Die Onlineumfrage der Jungen DIVI zum Ankommen in der Intensivmedizin zeichnete ein gemischtes Bild von der Einarbeitung und Arbeitszufriedenheit junger Fachkräfte in der deutschen Intensivmedizin.

Grundsätzlich muss gemäß den Umfrageergebnissen konstatiert werden, dass die Einarbeitung optimierungsbedürftig ist. Dies schlägt sich insbesondere darin nieder, dass sich 47 % der Befragten durch die Einarbeitung nicht ausreichend auf die Tätigkeit vorbereitet und 42 % im beruflichen Alltag „oft“ überfordert fühlten. Dass bekannte Methoden zur Strukturierung der Einarbeitung (Einarbeitungskonzept, Clinical Mentoring) weitgehend nicht genutzt bzw. nicht ausreichend umgesetzt werden, wenngleich ihre Wirksamkeit wissenschaftlich belegt werden konnte [3, 7], zeigt aus Sicht der Autor:innen erheblichen Nachbesserungsbedarf. Auch die teilweise fehlende Verfügbarkeit von Simulationstrainings und strukturierten Vorbereitungskursen als vielfältig evidente Maßnahme zur Verbesserung der individuellen und Teamperformance sowie Steigerung der Patient:innensicherheit erscheint verbesserungsbedürftig [6].

Auffällig sind die offenbarten Berufsgruppenunterschiede. Sicherlich liegt darin jedoch auch die Chance, voneinander zu lernen, wie die Einarbeitung neuer Mitarbeitender verbessert werden kann. In den neuen DIVI-Strukturempfehlungen aus dem Jahr 2022 wird dem besonderen Stellenwert der Einarbeitung neuer Mitarbeitender detailliert Rechnung getragen [8]. Die empfohlene 3‑monatige Einarbeitungsphase steht in deutlichem Kontrast zur berichteten mittleren Einarbeitungsdauer in der Studienkohorte (7 respektive 30 Tage Einarbeitungszeit bei Ärzt:innen bzw. Pflegefachpersonen). Hier sind dringend Nachbesserungen in der gelebten Praxis erforderlich und es muss der klare Auftrag formuliert werden, ausreichende Ressourcen für die Einarbeitung junger Fachkräfte zur Verfügung zu stellen bzw. vorzuhalten. Die Teilnehmenden äußerten insbesondere den Wunsch nach einem:einer fest zugeordneten Ansprechpartner:in in der Einarbeitungszeit. Die Umsetzung würde zwar arbeitgeberseitig einen koordinativen Mehraufwand bedeuten, aber keine großen monetären Mittel erfordern. Dass sich einige der Teilnehmenden einen verstärkten Fokus auf theoretische Inhalte und Lernzielkontrollen wünschen, zeugt davon, dass auch die zunehmende Verfügbarkeit von kostenfreien Onlineressourcen (wie beispielsweise Free Open Access Medical Education, FOAMed; [5]) neben den Kenntnissen aus Studium, Ausbildung oder Fachlehrbüchern keine ausreichende Wissensgrundlage für den Start auf der Intensivstation sicherstellt. Auch wenn ein gewisser Grad an selbstständiger theoretischer Vorbereitung durchaus gefordert werden kann, sind einzuarbeitende Kolleg:innen dringend auf Strukturierung, Priorisierung und lokale Anpassung der teilweise unübersichtlichen Ressourcen durch die Einarbeitenden angewiesen. Hier sollte insbesondere eine individuelle Anpassung der Einarbeitungsinhalte an den Wissens- und Kompetenzstand erfolgen und vorab die gegenseitigen Erwartungen an den Einarbeitungsprozess beispielsweise in einem Gespräch ausreichend früh (z. B. 3 Monate) vor dem Start auf der Intensivstation kommuniziert und abgeglichen werden. Hierbei ergäbe sich auch die Möglichkeit, den Einzuarbeitenden klinikspezifische Lektüre oder allgemeine Lehrmedien zu empfehlen.

Erfreulicherweise hat in der Studienkohorte die weitgehend negativ eingeschätzte Einarbeitung nicht zu einer mangelhaften Gesamtzufriedenheit geführt. Wesentliche Garanten für gesunde Arbeitsatmosphäre, wie ausreichend empfundener Lernzuwachs sowie gute interprofessionelle Zusammenarbeit, werden in der Studienkohorte zumeist als gegeben angesehen. Weniger zufriedenstellend sind jedoch die Aussagen zur Zufriedenheit mit den Arbeitsbedingungen. Nur eine Minderheit (27 %) kann die Erfüllung der im Sozialgesetzbuch V geforderten Merkmale „ausreichend und zweckmäßig“ in Bezug auf die Rahmenbedingungen bejahen. Die Politik ist gefordert, die Ausstattung der Intensivmedizin substanziell zu verbessern, sodass eine hochqualitative Versorgung unserer Intensivpatient:innen auch in Zukunft gesichert werden kann. Auch wenn Karrieremöglichkeiten teilweise recht heterogen eingeschätzt werden, sehen über 40 % der Befragten eine mehrjährige berufliche Zukunft in der Intensivmedizin.

Eine Limitation der Generalisierbarkeit unserer Aussagen ist die fehlende Repräsentativität der Stichprobe. Die Umfrageteilnahme wurde primär über DIVI-assoziierte Medien und mithilfe der Veröffentlichungen der DIVI beworben, sodass davon auszugehen ist, dass primär Teilnehmende angesprochen wurden, die sich überdurchschnittlich für die Intensivmedizin interessieren. Intensivmediziner:innen und Pflegefachpersonen in repräsentativer Art und Weise zu befragen, wird durch weitgehende Unklarheit über die Charakteristika der Grundgesamtheit sowie fehlende zentrale Registrierung (wie diese in anderen europäischen Staaten verfügbar ist) erschwert. Unsere Methodik folgte dem Ziel, in der Intensivmedizin Tätige und Engagierte zu befragen, führte jedoch zu einer Überpräsentation von Arbeitnehmer:innen von großen maximalversorgenden und universitären Kliniken. Hieraus ergibt sich eine Unschärfe in Bezug auf die Übertragbarkeit der Ergebnisse auf Intensivstationen in kleineren Krankenhäusern.

Universitäre Intensivmedizin unterscheidet sich unter anderem partiell in Umfang, Komplexität und Erkrankungsschwere der Patienten, zudem nehmen diese Kliniken traditionell einen Großteil des intensivmedizinischen Lehrauftrags wahr. Bettseitiger Unterricht wird dabei nicht selten durch junge Fachkräfte unterstützt. Betrachtet man unter dieser Prämisse die erlebte Unsicherheit der jungen Kolleg:innen, darf aus unserer Sicht die Frage gestellt werden, wie diese jungen Fachkräfte authentisch Begeisterung und Interesse für die Intensivmedizin bei Studierenden und Auszubildenden wecken sollen, wenn sie selbige zu Beginn ihrer Tätigkeit selbst nicht erleben. Es ist folglich nicht auszuschließen, dass eine unzureichende Einarbeitung junger Fachkräfte indirekt auch negative Effekte auf die nachfolgende Generation angehender Fachkräfte haben könnte. Dem gilt es, durch eine strukturierte und umfassende Einarbeitung vorzubeugen.

### Ausblick

Für eine zufriedenstellende Einarbeitung fehlte gemäß unserer Umfrage durchschnittlich sowohl die nötige Zeit als auch die erforderliche Struktur, um den jungen Fachkräften zum Start auf der Intensivstation eine ausreichende Vorbereitung zu ermöglichen. Dennoch kann eine grundsätzliche Zufriedenheit mit der intensivmedizinischen Tätigkeit festgehalten werden. Es ist Aufgabe der Krankenhäuser, Fachgesellschaften und Politik, Rahmenbedingungen zu schaffen, die dazu beitragen, diese motivierten Fachkräfte auch langfristig in der Intensivmedizin zu halten. Nur so kann nachhaltig die Versorgungssicherheit und -qualität in deutschen Intensivstationen gewährleistet werden. Eine entscheidende Weichenstellung erfolgt bereits bei der Einarbeitung auf der Intensivstation, die ausreichend, strukturiert und begleitet erfolgen sollte. Wir sehen hierbei eine große Chance, Fachkräfte zum langfristigen Bleiben in der Intensivmedizin zu begeistern.

## Fazit für die Praxis

- Einarbeitung auf der Intensivstation erfüllt sowohl zeitlich als auch strukturell nicht die Empfehlungen und, beinahe noch wichtiger, nicht die Erwartungen der einzuarbeitenden Fachkräfte, was in Unsicherheit und Überforderung resultiert.
- Für die nachhaltige Sicherung der Versorgungssicherheit und -qualität intensivmedizinischer Patient:innen in Deutschland gilt es, strukturierte Einarbeitungskonzepte mit ausreichendem Umfang und Inhalt bundesweit und verbindlich zu etablieren.
- Junge Fachkräfte sind mit ihrer Tätigkeit sowie ihrem Lernerfolg im intensivmedizinischen Kontext grundsätzlich zufrieden und sehen generell eine berufliche Perspektive in diesem Bereich.

### Supplementary Information





## References

[1] Bitzinger D, Raspe M, Schulte K, Larmann J (2017) Evaluation der Arbeits- und Weiterbildungsbedingungen im Fachgebiet Anästhesiologie in Deutschland – Ergebnisse einer bundesweiten Befragung. Anästh Intensivmed 58:429–440

[2] Ernst A-K, Zupanic M, Ellrichmann G, Biesalski A-S (2022). Germany-wide evaluation of residency in neurological intensive care medicine. BMC Med Educ.

[3] Feld F, Sopka S, Stieger L (2015). Innovationen in der intensivmedizinischen Weiterbildung – Das Aachener „Clinical-Mentor-Konzept“ in der operativen Intensivmedizin und Intermediate Care. Anästh Intensivmed.

[4] Karagiannidis C, Janssens U, Kluge S (2021). Intensivstationen: Ein Drittel der Betten ist gesperrt. Dtsch Arztebl Int.

[5] Olusanya O, Day J, Kirk-Bayley J, Szakmany T (2017). Free Open Access Med(ical edu)cation for critical care practitioners. J Intens Care Soc.

[6] Seam N, Lee AJ, Vennero M, Emlet L (2019). Simulation training in the ICU. Chest.

[7] Spacek M, Prautzsch C, Mehrholz J, Eberlein-Gonska M (2022). Positive Effekte eines Einarbeitungs- und Weiterbildungscurriculums am Beispiel der interdisziplinären Intensivstation. Med Klin Intensivmed Notfmed.

[8] Waydhas C, Riessen R, Markewitz A (2023). DIVI-Empfehlung zur Struktur und Ausstattung von Intensivstationen 2022 (Erwachsene). Med Klin Intensivmed Notfmed.

