# Supplementary material for: Onboarding of young professionals in the intensive care unit: Nationwide online survey by young DIVI – an initiative of the German interdisciplinary association for intensive and emergency medicine (DIVI)
Source: Med Klin Intensivmed Notfmed. 2023 Sep 27;119(4):302–8. [Article in German] doi: 10.1007/s00063-023-01067-y (PMC11058678; doi:10.1007/s00063-023-01067-y)
Supplement: Supplementary file 1 [file 63_2023_1067_MOESM1_ESM.docx]

**Supplement**

**Einarbeitung junger Fachkräfte auf der Intensivstation**

Eine bundesweite Onlineumfrage der Jungen DIVI, einer Initiative der Deutschen Interdisziplinären Vereinigung für Intensiv- und Notfallmedizin

***Onboarding of young professionals in the ICU***

*A nationwide online survey by young DIVI, an initiative of the German German Interdisciplinary Association for Intensive and Emergency Medicine (DIVI)*

David Josuttis^1*^, Frida Regner^2^, Teresa Deffner^3^, Diana Freund^4^, Felix Freund^5^, Celina Cornelius^6^, Angelina Beer^2^, Aileen Spieckermann^7^, Matthias Manfred Deininger^8^

^1^ Klinik für Anästhesiologie, Intensiv- und Schmerzmedizin, BG-Klinikum Unfallkrankenhaus Berlin

^2^ Klinik und Poliklinik für Kinder- und Jugendmedizin, Universitätsklinikum Carl Gustav Carus Dresden

^3^ Klinik für Anästhesiologie und Intensivmedizin, Universitätsklinikum Jena

^4^ Klinik für Anästhesie, Intensiv-, Notfall- und Schmerzmedizin, Universitätsmedizin Greifswald

^5^ Offshore Rescue and Medical Service, Die Johanniter Unfall-Hilfe e.V.

^6^ Klinik für Anästhesiologie, Universitätsklinikum Heidelberg

^7^ Chirurgische Universitätsklinik und Poliklinik, BG Universitätsklinikum Bergmannsheil

^8^ Klinik für Anästhesiologie und Klinik für Operative Intensivmedizin und Intermediate Care, Uniklinik RWTH Aachen

* Korrespondierender Autor: David.Josuttis@ukb.de

**Material S1: Fragebogen mit erklärenden Einleitungstexten**

*Die Deutsche interdisziplinäre Vereinigung für Intensiv- und Notfallmedizin (DIVI) hat 2022 eine neue Initiative gegründet, die Junge DIVI.*

*Als Junge DIVI verstehen wir uns als Interessenvertretung junger Fachkräfte in der Intensiv- und Notfallmedizin und als Ansprechpartner für interessierte junge Menschen an eben diesen Fachbereichen. Unsere Aufgabe ist es, sowohl innerhalb der DIVI, als auch nach außen die Perspektive junger Fachkräfte zu vertreten.*

*Aus diesem Grund wollen wir Umfragen nutzen, um ganz gezielt bei euch, der Interessengemeinschaft nachzufragen.*

*Die Umfrage ist die allererste der Jungen DIVI. Wir nutzen diesen Aufschlag, um euch kennenzulernen und wollen die Themen Einarbeitung, Skillraining, Mentoring und Intensiv-Vorbereitungskurs in den Fokus rücken. Kurz gesagt, es geht um das „Ankommen in der Intensivmedizin“. Wir wollen wissen, wer kennt und nutzt welche dieser Modelle und sind sie hilfreich? Außerdem möchten wir gerne einen Eindruck von der generellen Arbeitszufriedenheit junger Fachkräfte in der Intensiv- und Notfallmedizin bekommen.*

*Wenn du als Arzt/Ärztin oder Pflegekraft in der Intensivmedizin tätig bist oder in den letzten Jahren tätig warst, würden wir uns freuen, wenn du dir die Zeit nimmst, unsere Fragen zu beantworten.*

*Die Umfrage ist in ca. 10 min zu absolvieren.*

*Die Teilnahme ist freiwillig. Nach Eingabe der Daten werden diese nur zur Beantwortung der Fragestellung genutzt und vollständig anonymisiert gespeichert und verarbeitet.*

*Bei Rückmeldungen oder Fragen zum Thema oder der Umfrage, wende dich jederzeit gerne an umfrage.jungedivi@gmail.com.*

*Die Ergebnisse werden auf dem DIVI22-Kongress in Hamburg (30.11-02.12.) vorgestellt und diskutiert.*

*Vielen Dank für eure Zeit und euer Interesse.*

**Soziodemografische Daten**

Geschlecht

- - Non-binär
  - Weiblich
  - Männlich
  - Keine Angaben

Alter

- < 25 Jahre
- 25-35 Jahre
- >35 Jahre
- Keine Angaben

Ich werde / ich bin:

- Pflegende/r

Mein höchster Bildungsabschluss:

- Allgemeinbildende Schule (Auszubildende/r)
- Abgeschlossene Berufsausbildung in der Pflege
- Abgeschlossene Fachweiterbildung in der Pflege
- Akademischer Abschluss in der Pflege (Bachelor, Master, Vergleichbares)
- Sonstiges: (Freitexteingabe)

Ich werde / ich bin:

- Ärztin / Arzt

Mein höchster Bildungsabschluss:

- Allgemeinbildende Schule (Medizinstudent/IN)
- Staatsexamen
- Facharztprüfung
- Zusatzweiterbildung / Zusatzbezeichnung
- Sonstiges: (Freitexteingabe)

Ich bin…

- …aktuell auf Intensivstation tätig.
- …nicht mehr auf Intensivstation tätig.
- …in der Zukunft auf Intensivstation tätig oder nicht sicher, ob ich jemals auf einer Intensivstation tätig sein werden. *(Umfrage endet, Hinweistext)*

*Leider endet die Umfrage für dich an dieser Stelle. Wir bedanken uns für dein Interesse und hoffen du bleibst uns für zukünftige Umfragen erhalten.*

Für meinen Start auf Intensivstation wünsche ich mir….

- …eine/n feste/n Ansprechpartner/in.
- …regelmäßige Anleitungen.
- …die Möglichkeit an fallbezogenen Simulationstrainings teilzunehmen.
- …zuvor einen Vorbereitungskurs absolvieren zu können.
- Sonstiges: (Freitext)

Meine Tätigkeit auf Intensivstation habe ich begonnen…

- …2022 – 2020.
- …2019 – 2017.
- …vor 2017.

Ich arbeite im Fachbereich:

- Anästhesie
- Interdisziplinär
- Innere Medizin
- Chirurgie
- Neurologie / Neurochirurgie
- Herzchirurgie
- Pädiatrie
- Sonstige: (Freitext)
- Keine Angaben

Die Intensivstation, auf der ich tätig bin / war, führt:

- < 10 Patientenbetten
- 10-20 Patientenbetten
- 21-30 Patientenbetten
- > 30 Betten

Versorgungsstufen / Träger des Krankenhauses an dem ich tätig bin (ggf. Mehrfachnennung):

- Fachkrankenhaus
- Klinik der Grundversorgung
- Klinik der Regelversorgung
- Klinik der Schwerpunktversorgung
- Klinik der Maximalversorgung
- Universitätsklinikum
- Sonstiges: (Freitext)
- Keine Angaben

Ich bin DIVI – Mitglied / Mitglied der Jungen DIVI

- Ja
- Nein

**Einarbeitungskonzept**

Unter Einarbeitungskonzept verstehen wir in diesem Kontext einen strukturierten, schriftlich verfügbaren Plan. Anhand dessen können sich Mitarbeiter/Innen mit der neuen Arbeitsumgebung schnell vertraut machen und finden alle relevanten Informationen zur neuen Tätigkeit in übersichtlicher Art und Weise.

Er definiert, welche Tätigkeiten, ab welchem Zeitpunkt eigenverantwortlich übernommen werden sollen. Im Folgenden geht es um die Intensivstation auf der du eingearbeitet wurdest.

Gab es / gibt es auf der Intensivstation, auf der du eingearbeitet wurdest ein Einarbeitungskonzept / Clinical Mentor Konzept?

- Ja

Wurdest du damit eingearbeitet?

- - Ja

War es hilfreich für das Ankommen auf ITS?

- - - Überhaupt nicht hilfreich
    - Eher nicht hilfreich
    - Teils nicht hilfreich / teils hilfreich
    - Eher hilfreich
    - Sehr hilfreich
  - Nein
  - Teilweise, weil… (Mehrfachnennung möglich)
    - …nicht ausreichend personelle Ressourcen vorhanden sind / waren für die Einarbeitung.
    - …nicht ausreichend zeitliche Ressourcen vorhanden sind / waren für die Einarbeitung.
    - …das vorgehaltene Konzept auf dem Papier existiert, aber nicht strukturiert angewendet wird / angewandt wurde, trotz ausreichender personeller und zeitlicher Ressourcen.
    - Sonstige: (Freitext)
- Nein

Hättest du es dir gewünscht?

- Ja
- Nein
- Keine Angaben

**Clinical Mentor / Praxisanleitung**

Ein erfahrener Kollege (=“Clinical Mentor“) hat sich um die Optimierung von Einarbeitung und Weiterbildung hauptamtlich gekümmert und / oder wurde dafür sogar freigestellt. Die Mentorin / der Mentor war verantwortlich für die strukturierte Einführung und die praktisch-klinische Anleitung. Während der Einarbeitungsphase kam es immer wieder zu Anleitungssituationen durch einen festen Ansprechpartner / Praxisanleiter.

Wurdest du während deiner Einarbeitung durch einen (Clinical) Mentor und / oder Praxisanleiter betreut?

- Ja

War es hilfreich für das Ankommen auf ITS?

- - - Überhaupt nicht hilfreich
    - Eher nicht hilfreich
    - Teils nicht hilfreich / teils hilfreich
    - Eher hilfreich
    - Sehr hilfreich
- Nein

Hättest du es dir gewünscht?

- Ja
- Nein
- Keine Angaben
- Teilweise, weil… (Mehrfachnennung möglich)
  - - …mir kein feste/r Ansprechpartner/in zugeordnet war.
    - …die tatsächliche Einarbeitungszeit, explizite Zeit mit dem Mentor, war viel kürzer, als geplant / vereinbart.
    - ...ich eine/n festen Ansprechpartner/in hatte, aber es zu keinen Anleitungen kam.
    - Sonstige: (Freitext)

**Simulationskurs / fallbezogenes Simulationstraining**

Simulation ist eine Technik zum Üben und Erlernen, die auf viele verschiedene Disziplinen und Auszubildende angewendet werden kann. Über Simulationskurse / fallbezogenes Simulationstraining werden Wissen und Fähigkeiten sowie Fertigkeiten ausgebaut, es dient der Patienten- und Anwendersicherheit.

Charakteristisch ist die realitätsnahe Darstellung von Szenarien in möglichst echten Umgebungen und mit typisch zusammengestellten Teams.

Gab es / gibt es im Rahmen der Einarbeitung einen Simulationskurs bzw. regelmäßige fallbezogene Simulationstrainings und hast du daran teilgenommen?

- Ja

War es hilfreich für das Ankommen auf ITS?

- - - Überhaupt nicht hilfreich
    - Eher nicht hilfreich
    - Teils nicht hilfreich / teils hilfreich
    - Eher hilfreich
    - Sehr hilfreich
- Nein

Hättest du es dir gewünscht?

- Ja
- Nein
- Keine Angaben

Welche Gründe standen einer Teilnahme entgegen?

- Der Zeitpunkt des Angebotes war für mich, im Kontext meiner Einarbeitung nicht optimal (zu zeitig, zu spät).
- Es fand außerhalb der Arbeitszeit statt.
- Ich wurde nicht dafür freigestellt.
- Es gab (zu hohe) Teilnahmegebühren.
- Die Zusammensetzung der Teilnehmer empfand ich als unpassend.
- Ich sah mich nicht als Zielgruppe des Kurses.
- Ich war nicht verpflichtet teilzunehmen.
- Sonstiges: (Freitext)

**Intensiv-Vorbereitungskurs**

Ein Intensiv-Vorbereitungskurs bereitet junge Pflegende und Ärztinnen / Ärzte auf ihre zukünftige Tätigkeit auf Intensivstation vor. Durch eine Kombination aus Vorlesungen, Kleingruppenarbeit und Skill-Stationen (Hands-On Trainings) wird das intensivmedizinische Wissen erweitert und etwaige Ängste im Vorfeld abgebaut.

Ich weiß, dass es Intensiv-Vorbereitungskurse in Deutschland gibt?

- Ja

Hast du an einem (mehreren) teilgenommen?

- - Ja

War es hilfreich für dein Ankommen auf ITS?

- - - Überhaupt nicht hilfreich
    - Eher nicht hilfreich
    - Teils nicht hilfreich / teils hilfreich
    - Eher hilfreich
    - Sehr hilfreich
  - Nein

Welche Faktoren standen einer Teilnahme entgegen?

- Der Zeitpunkt des Angebotes war für mich, im Kontext meiner Einarbeitung nicht optimal (zu zeitig, zu spät).
- Es fand außerhalb der Arbeitszeit statt.
- Ich wurde nicht dafür freigestellt.
- Es gab (zu hohe) Teilnahmegebühren.
- Die Zusammensetzung der Teilnehmer empfand ich als unpassend.
- Ich sah mich nicht als Zielgruppe des Kurses.
- Ich war nicht verpflichtet teilzunehmen.
- Sonstiges: (Freitext)
- Nein

**Persönliches Fazit zur Einarbeitung**

Ich fühl(t)e mich durch meine Einarbeitung ausreichend auf meine Tätigkeit in der Intensivmedizin vorbereitet.

- - - Stimme überhaupt nicht zu
    - Stimme nicht zu
    - Stimme weder zu noch lehne ich ab
    - Stimme zu
    - Stimme voll und ganz zu

Was hätte es gebraucht, damit mein Ankommen auf der Intensivstation einfacher verlaufen wäre?

- Freitext

**Zufriedenheit**

Im Folgenden geht es um deine allgemeine Zufriedenheit mit deiner beruflichen Tätigkeit.

Ich bin mit meiner Tätigkeit in der Intensivmedizin grundsätzlich zufrieden.

- - - Stimme überhaupt nicht zu
    - Stimme nicht zu
    - Stimme weder zu noch lehne ich ab
    - Stimme zu
    - Stimme voll und ganz zu

Ich erhalte konstruktives Feedback.

- - - Stimme überhaupt nicht zu
    - Stimme nicht zu
    - Stimme weder zu noch lehne ich ab
    - Stimme zu
    - Stimme voll und ganz zu

Ich bin mit meinem Lernzuwachs im Berufsalltag zufrieden.

- - - Stimme überhaupt nicht zu
    - Stimme nicht zu
    - Stimme weder zu noch lehne ich ab
    - Stimme zu
    - Stimme voll und ganz zu

Ich bin mit meinen Karrieremöglichkeiten/ beruflichen Weiterentwicklungsmöglichkeiten zufrieden.

- - - Stimme überhaupt nicht zu
    - Stimme nicht zu
    - Stimme weder zu noch lehne ich ab
    - Stimme zu
    - Stimme voll und ganz zu

Die Arbeitsbedingungen (Infrastruktur, materielle und personelle Ausstattung) sind mindestens ausreichend und zweckmäßig.

- - - Stimme überhaupt nicht zu
    - Stimme nicht zu
    - Stimme weder zu noch lehne ich ab
    - Stimme zu
    - Stimme voll und ganz zu

Ich bin mit der interprofessionellen Zusammenarbeit in meiner Tätigkeit zufrieden.

- - - Stimme überhaupt nicht zu
    - Stimme nicht zu
    - Stimme weder zu noch lehne ich ab
    - Stimme zu
    - Stimme voll und ganz zu

Ich habe häufig das Gefühl, überfordert zu sein.

- - - Stimme überhaupt nicht zu
    - Stimme nicht zu
    - Stimme weder zu noch lehne ich ab
    - Stimme zu
    - Stimme voll und ganz zu

Ich kann mir vorstellen langfristig (>3 Jahre) in der Intensivmedizin tätig zu sein.

- - - Stimme überhaupt nicht zu
    - Stimme nicht zu
    - Stimme weder zu noch lehne ich ab
    - Stimme zu
    - Stimme voll und ganz zu

Ich kann mir vorstellen, aktiv bei der jungen DIVI mitzuwirken.

- - - Stimme überhaupt nicht zu
    - Stimme nicht zu
    - Stimme weder zu noch lehne ich ab
    - Stimme zu
    - Stimme voll und ganz zu

**Tabelle S2: Demografische Ergebnisse - Übersicht über die teilnehmende Kohorte**

| Kategorie |  | Ärzt:innen (n=370) | Pflegefachpersonen (n=184) |
| --- | --- | --- | --- |
| Geschlecht | männlich | 174 (48,1%) | 59 (32,2%) |
|  | weiblich | 185 (51,2%) | 122 (66,7%) |
|  | non-binär | 2 (0,6%) | 2 (1,1%) |
| Alter | <25 Jahre | 6 (1,7%) | 23 (12,8%) |
|  | 25-35 Jahre | 229 (63,4%) | 77 (42,8%) |
|  | >35 Jahre | 126 (34,9%) | 80 (44,4%) |
| Tätigkeitsbeginn auf der Intensivstation | 2020-2022 | 179 (54,2%) | 28 (17,3%) |
|  | 2017-2019 | 69 (20,9%) | 30 (18,5%) |
|  | vor 2017 | 82 (24,9%) | 104 (64,2%) |
| Fachrichtung der Intensivstation | Anästhesiologie | 212 (63,9%) | 24 (14,6%) |
|  | Chirurgie | 12 (3,6%) | 5 (3%) |
|  | Gefäßchirurgie | 1 (0,3%) | 0 |
|  | Herz- und Thoraxchirugie | 2 (0,6%) | 19 (11,6%) |
|  | Innere Medizin | 61 (18,4%) | 18 (11,0%) |
|  | Interdisziplinär | 5 (1,5%) | 56 (34,1%) |
|  | Neonatologie/Pädiatrie | 0 | 1 (0,6%) |
|  | Neurologie/ Neurochirurgie | 19 (5,7%) | 15 (9,1%) |
|  | Orthopädie/ Unfallchirurgie | 4 (1,2%) | 11 (6,7%) |
|  | Plastische Chirurgie | 14 (4,2%) | 3 (1,8%) |
|  | Sonstiges | 0 | 5 (4,3%) |
| Größe der Intensivstation | <10 Betten | 22 (6,6%) | 23 (14,1%) |
|  | 10-20 Betten | 209 (63,0%) | 109 (66,9%) |
|  | 21-30 Betten | 66 (19,9%) | 25 (15,3%) |
|  | >30 Betten | 35 (10,5%) | 6 (3,7%) |
| Versorgungsstufe des Krankenhauses | Fachkrankenhaus | 3 (0,8%) | 12 (6,5%) |
|  | Grundversorgung | 11 (2,9%) | 13 (7,0%) |
|  | Regelversorgung | 40 (10,6%) | 29 (15,6%) |
|  | Schwerpunktversorgung | 36 (9,5%) | 12 (6,5%) |
|  | Maximalversorgung | 100 (26,5%) | 53 (28,5%) |
|  | Universitätsklinikum | 187 (49,6%) | 67 (36,0%) |
| Bundesland | Baden-Württemberg | 35 (10,6%) | 18 (11,1%) |
|  | Bayern | 43 (13,0%) | 19 (11,7%) |
|  | Berlin | 61 (18,5%) | 14 (8,6%) |
|  | Brandenburg | 10 (3,0%) | 0 |
|  | Bremen | 2 (0,6%) | 0 |
|  | Hamburg | 12 (33,6%) | 8 (4,9%) |
|  | Hessen | 23 (7,0%) | 7 (4,3%) |
|  | Mecklenburg-Vorpommern | 3 (0,9%) | 0 |
|  | Niedersachsen | 21 (6,4%) | 19 (11,7%) |
|  | Nordrhein-Westfalen | 86 (26,1%) | 52 (32,1%) |
|  | Rheinland-Pfalz | 2 (0,6%) | 5 (3,1%) |
|  | Saarland | 1 (0,3%) | 0 |
|  | Sachsen | 13 (3,9%) | 14 (8,6%) |
|  | Sachsen-Anhalt | 3 (0,9%) | 1 (0,6%) |
|  | Schleswig-Holstein | 15 (4,5%) | 4 (2,5%) |
|  | Thüringen | 0 | 1 (0,6%) |

**Ausbildungsstand der teilnehmenden Ärzt:innen**

| Qualifikation | Medizinstudierende | 7 (1,9%) |
| --- | --- | --- |
|  | in Facharztweiterbildung | 220 (61,3%) |
|  | Fachärztin/ Facharzt | 132 (36,8%) |
| Im Besitz der Zusatzweiterbildung oder Zusatzbezeichnung (Mehrfachantwort möglich) | Notfallmedizin | 159 (43,0%) |
|  | Intensivmedizin | 71 (19,2%) |
|  | Klinische Akut- und Notfallmedizin | 8 (2,2%) |
|  | Keine der genannten | 182 (49,2%) |

**Ausbildungsstand der teilnehmenden Pflegefachpersonen**

| Qualifikation | Auszubildende:r | 1 (0,6%) |
| --- | --- | --- |
|  | Berufsausbildung | 52 (30,6%) |
|  | akademischer Abschluss | 29 (17,1%) |
|  | Fachweiterbildung | 90 (53,0%) |

Die prozentualen Angaben beziehen sich auf die Teilnehmendenzahl der jeweilige Berufsgruppe, die die Frage beantwortet haben.
